# Evaluation of CD44+/CD24- and Aldehyde Dehydrogenase Enzyme Markers in Cancer Stem Cells as Prognostic Indicators for Triple-Negative Breast Cancer

**DOI:** 10.7759/cureus.28056

**Published:** 2022-08-16

**Authors:** Lisbeth Escudero Mendez, Mirra Srinivasan, Ranim K Hamouda, Baba Ambedkar, Hadia Arzoun, Isra Sahib, Jack Fondeur, Lubna Mohammed

**Affiliations:** 1 Pathology, California Institute of Behavioral Neurosciences & Psychology, Fairfield, USA; 2 Internal Medicine, California Institute of Behavioral Neurosciences & Psychology, Fairfield, USA

**Keywords:** cancer stem cells, triple-negative breast cancer, ladh or ladh1, cd24-, cd44+, prognosis

## Abstract

Triple-negative breast cancer (TNBC) has been extensively studied not just for its aggressive behavior but also to understand its complex molecular nature. This type of heterogeneous tumor shows no expression of estrogen receptor (ER) or progesterone receptor (PR) and does not express the HER2 gene, and often these tumors are high grade with distinct histological groups. The basal-like subtype is most commonly related to the TNBC type of neoplasms; it can be further classified according to Lehmann and Burstein expert’s criteria. TNBC is related to breast stem cell markers such as CD44+/CD24- and high levels of enzyme aldehyde dehydrogenase (ALDH), which have been shown to possess stem cell features that are involved in differentiation, vascular invasion, tumorigenesis, and metastatic potential. CD44+/CD24- and high levels of ALDH have significance as markers as well as indicators of poor prognosis in TNBC. The databases used in this review are PMC, PubMed, and Google Scholar.

## Introduction and background

The World Health Organization (WHO) declared in 2020 that “2.3 million women were diagnosed with breast cancer with 685,000 deaths globally. As of the end of 2020, there were 7.8 million women alive who were diagnosed with breast cancer in the past five years, making it the world’s most prevalent cancer” [1].

Around 10-24% of the breast cancer population constitutes invasive breast cancer with triple-negative breast cancer (TNBC) markers, which are tumors that do not display estrogen receptor (ER) or progesterone receptor (PR), and are characterized by non-overexpression of the HER2 gene; they are often high-grade tumors with distinct histological groups [2]. The TNBC arises mainly in young premenopausal African or African American women and tends to behave aggressively with metastatic potential. Bones and the brain are the most common metastatic sites [2,3]. Women diagnosed with the TNBC phenotype are prone to have a higher recurrence rate, a short disease-free interval, and reduced overall survival (OS), particularly in the absence of appropriate treatment [4]. TNBC has been basically classified into two groups: the basal-like subtype that accounts for 70% of TNBCs (showing basal markers) and the nonbasal subtype [5,6].

Breast neoplasm is phenotypically comprised of different types of cells; however, the cells that induce tumor progression are deemed equivocal. Although native cells can evolve through alterations, a specific group of cells has shown the ability to self-renew and differentiate [7-9]. This very heterogeneous population is labeled breast cancer stem cells (BCSCs) [8]. BCSCs have been studied for their self-renewal features, which facilitate tumoral formation and progression [10]. Also, it has been proposed that BCSCs are capable of chemotherapy resistance and tumor recurrence [11,12]. The heterogeneity of these cells in the neoplasm has been studied with obscure results. Consequently, this hypothesis was investigated to navigate breast cancer heterogeneity to tumor recurrence [13]. Moreover, it has been stated that BCSCs can generate drug resistance, which leads to neoplasm recurrence or metastasis [14]. Stemness markers have been studied to identify BCSC, such as CD44/CD24 expression lines, which have shown a significant association with distant metastatic breast cancer subtypes for TNBCs [15]. The CD44+CD24-/low phenotype has been associated with a poor prognosis in TNBC patients [16].

**Figure 1:**
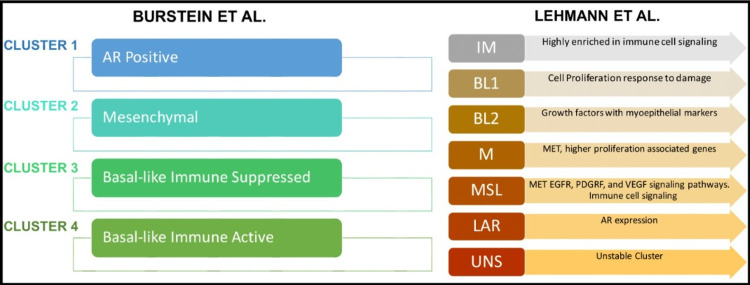
TNBC classification according to Burstein et al. and Lehmann et al. Lehmann et al. in 2011 divided TNBC into the following six molecular subgroups displaying unique genetic expression and ontologies: immunomodulatory (IM), mesenchymal (NI), mesenchymal stem-like (MSL), luminal androgen receptor (LAR), and two basal-like subtypes (BL1 and BL2). Burstein et al. in 2105 used DNA profiling to identify TNBC subgroups: cluster 1: LAR, cluster 2: mesenchymal (MES), cluster 3: basal-like immunosuppressed (BLIS), and cluster 4: basal-like immune-activated (BLIA). The two tables express similar information such as cluster 1 has Lehmann’s LAR neoplasms, cluster 2 has most of Lehmann’s mesenchymal stem-like and clusters 3 and 4 have Lehmann’s basal-like 1 and basal-like 2 neoplasms, and mesenchymal neoplasms are assigned to clusters 2 and 4 since they share common signaling pathways [17,18] The figure was created in Mind the Graph

TNBC has been categorized based on its tumor heterogeneity and diverse clinical outcomes into more specific subgroups by experts [17,18]. They used genomic expression profile (GEP) assays for molecular characterization of TNBC subtypes [17,18], as illustrated in Figure 1. CD44 is a compelling biomarker present in invasive breast cancer. It is a transmembrane glycoprotein of the hyaluronate receptor lineage, which is present in cell adhesion and extracellular matrix interaction dynamics [19]. CD44 can control other membrane tyrosine receptor roles. Moreover, it has been involved as a substitute marker for neoplasmic stem cells [20]. Evidence has shown that CD44 positive cells display the skill for tumoral initiation, propagation, and self-renewal [21].

CD24 is an adhesion molecule and binds to P-selectin, which is a protein expressed on thrombin-activated platelets and endothelial cells [22]. CD24 is present in various cell lines such as B-cell precursors, neutrophils, and neuronal tissue in addition to keratinocytes and renal tubular epithelium [23]. Also, its expression has been shown to initiate metastasis in vivo by regulating a system that promotes the expression of CD24 in mammary carcinoma cells and contributed to tumor cell proliferation, tumor cell binding to P-selectin, fibronectin, and other extracellular matrix components including motility and invasion [23]. This marker has been studied in tumorigenesis and the progression of several groups of tumors and as a prognosis marker of poor survival in breast neoplasms [20].

A small population of undifferentiated cells with properties of stemness, self-renewal, and regenerative ability is commonly seen in tumors [24]. These cancer cells behave like stem cells that comprise 0.1-1% of the tumor mass. Some of the first markers identified in cancer stem cells were CD44, CD24, and later an enzyme aldehyde dehydrogenase (ALDH or ALDH1); these markers were studied with fluorescence-activated cell sorting or immune selection methodologies [25].

**Figure 2:**
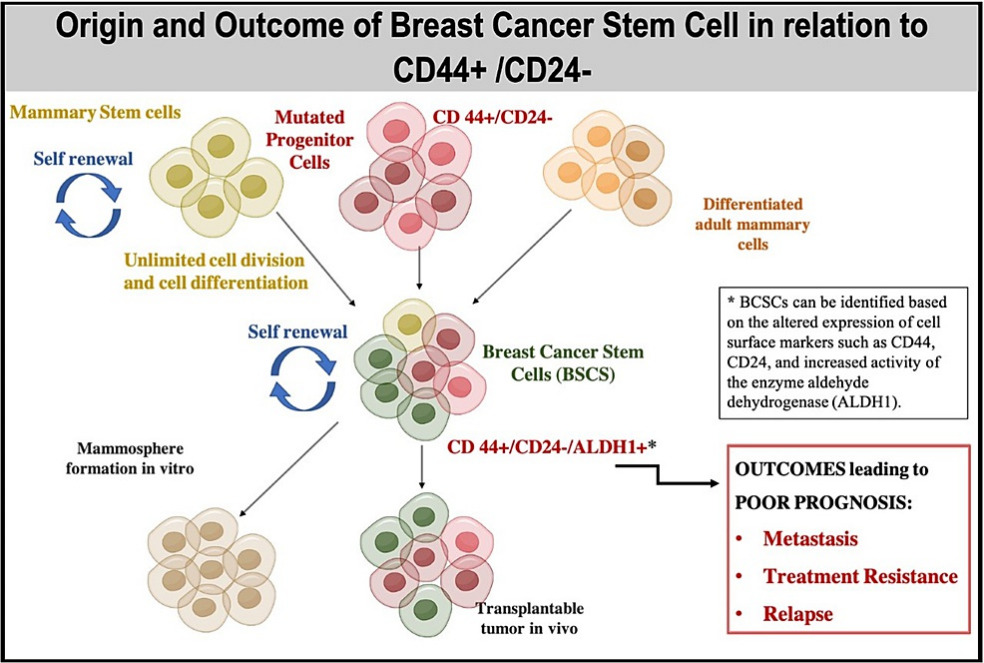
Breast cancer stem cells and tumorigenesis Figure Created in Mind the Graph

The theory of CSC is based on studies performed in human acute myeloid leukemia after a group of researchers observed that transplantation of a few leukemia cells formed new tumors in mice [26]. These subpopulations of CSC in the tumor are capable of self-regeneration and differentiation to become non-stem cancer cells that make up the tumor mass. Moreover, in BC, the tumor mass comes from BCSCs [27]. BCSCs express as CD44+/CD24-/Lin-. The origin of these cells is still a matter of debate, but there are two main theories: 1. normal aging stem cells accumulate mutations, and 2. mutations in a lineage-committed cell could gain stemness function [28]. Based on this, Figure 2 shows the abilities of BCSCs CD44+/CD24-, high activity of ALDH1 expression related to self-renewal, and unlimited cell division. Outcomes associated with the expression of these markers lead to factors associated with poor prognoses, such as metastasis, treatment resistance, and recurrence.

TNBC is an aggressive phenotype in breast neoplasms, and it is relevant to study the steps to improve the diagnosis with the advancement of molecular options and to search for the heterogeneity in certain groups of cells with properties as stem cells. The purpose of this article is to provide a better understanding of specific markers such as CD44+/CD24-low and ALDH enzymes in cancer stem cells in the prognosis of TNBC.

## Review

In this section, we discuss the fate of prognosis of TNBC in parallel to the presence of CD44+/CD24- and ALDH breast stem cell marker expression, the presence of metastatic regional lymph node and distant metastasis along with the heterogeneity in the cancer cell population, and contrasting results regarding CD44+/CD24-.

CD44+/CD24- marker phenotype and prognosis

Some studies (Table 1) in this review have confirmed the statistical significance between the presence of CD44+/CD24- and poor prognosis. In addition, these studies have analyzed the presence of BCSC markers in metastatic regional lymph nodes, lymphovascular invasion, and distance metastasis.

**Table 1:** Summary of the studies: prognosis according to CD44+/CD24- BCSC marker expression

Author	Year	Study population	Findings
Chekhun et al. [29]	2015	132 patients with BC stage 1-2	Statistical significance was found between the presence of CD44+/CD24- in metastatic regional lymph nodes along with overall survival (OS) in cases with basal-like and molecular subcategories
Li et al. [30]	2010	Immunohistochemistry of 103 cases of invasive micropapillary carcinoma (IMPC) and 94 cases of invasive ductal carcinoma (IDC)	CD44+/CD24- marker was observed in tumor cells in micropapillary and also in its stroma. Moreover, CD44+/CD24- was related to lymphovascular invasion, lymph node, and distant metastasis
Giatromanolaki et al. [31]	2010	139 cases of breast carcinomas (IDC) were investigated in paraffin sections using cell-like markers: (CD44, CD24), the ‘‘triple-state’’ (ER, PR, c-erb-B2), and angiogenesis (CD31)	Cases with CD44+/CD24- profile had a lower median age at onset of BC and showed a triple-negative state. They had a poor prognosis

Although not typical of all the important studies, Kim et al., in a retrospective study involving tissue microarray blocks of 643 cases of invasive breast carcinomas, have found that the CD44+/CD24- marker was taken as a positive prognostic subgroup in breast neoplasm [32], as explained in Table 2.

**Table 2:** Contrasting results for CD44+/CD24- and prognosis This study otherwise showed that CD44+/CD24- had positive prognostic results

Author	Year	Study population	Findings
Kim et al. [32]	2011	A retrospective study. Tissue microarray blocks of 643 cases of invasive breast carcinomas	CD44+/CD24- marker was taken as a positive prognostic subgroup in breast neoplasm

The number of positive lymph nodes is determined by different mechanisms such as phenotypic drift as a result of intratumoral heterogeneity [33] and dynamic plasticity of the tumor microenvironment [34]. These definitions are crucial in the understanding of BCSC behavior and the expression of markers useful for disease progression and treatment response. Al-Hajj et al. initiated the study of a different subgroup of cells within human breast carcinomas, which could recreate new neoplasms in immunocompromised mice. These tumor-starting cells were determined from the nontumorigenic cell population as they showed the CD44+/CD24- maker phenotype, which was associated with cancer stem cell features [20,35]. Since then, there has been more confirmation of CD44+/CD24- in BCSCs and their capacity for invasive features [36,37,29]. BCSC marker phenotype has shown a special preference to be expressed in the triple-negative basal molecular subgroup (44.8% versus 28.1% for luminal B subgroup) [29], and this study also found the presence of CD44+/CD24- in regional lymph nodes which were metastatic. Additionally, the CD44+/CD24- phenotype has been related to a poor prognosis [38]. It has been reported that those patients who presented CD44+/CD24- profile had a median age that was 10 years lower at the presentation of BC and had demonstrated a triple-negative state with poor prognosis [36]. Although not typical of all the important studies, Kim et al., in a retrospective study involving tissue microarray blocks of 643 cases of invasive breast carcinomas, have found that the CD44+/CD24- marker was taken as a positive prognostic subgroup in breast neoplasm [32]. In terms of OS rates, the analysis showed that women with CD44+/CD24- had a poor prognosis but a lower scale compared to the CD44-/CD24- phenotype [36]. Furthermore, the heterogeneity in BCSCs has been playing an important role in tumor features such as differentiation, vascular invasion, tumorigenesis, and metastatic potential [39].

Heterogeneity in breast cancer: intratumoral and intertumoral heterogeneity theory

Intratumoral Heterogeneity

**Figure 3:**
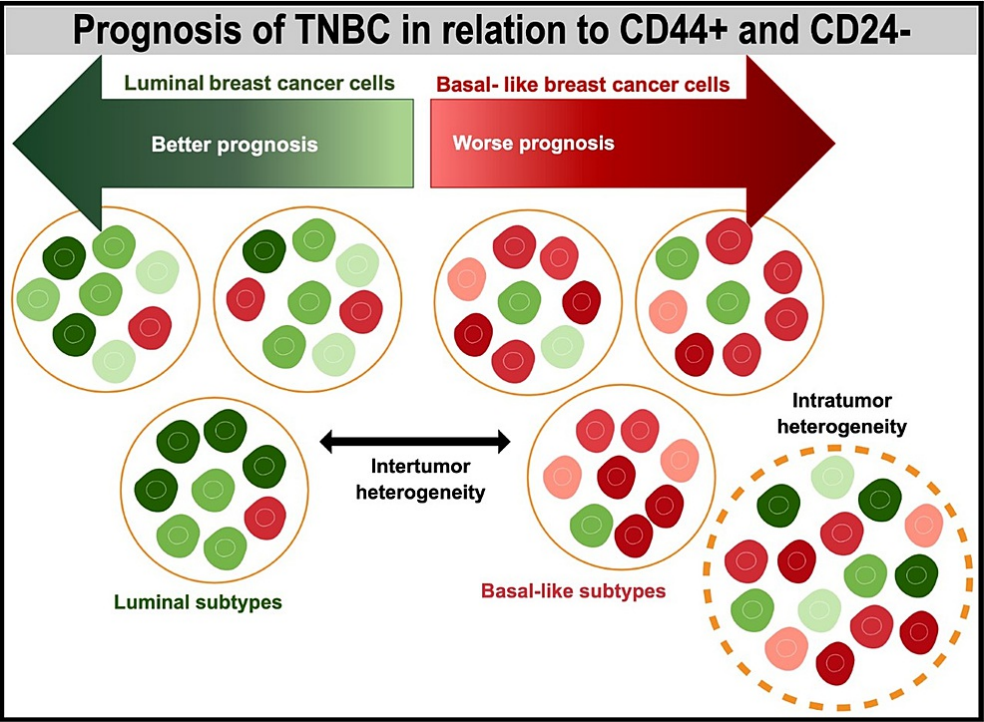
Heterogeneity in breast cancer: intratumoral and intertumoral heterogeneity theory and prognosis of TNBC Figure created in Mind the Graph

Intratumoral heterogeneity refers to a condition when a tumor is made up of different subgroups of cells. It has been described as a theoretical representation of intertumor and intratumor heterogeneity in breast cancer [34]. This figure represents multiple phenotypes and degrees of luminal and basal-like tumoral cells in the tumor called intratumor heterogeneity. Intertumor heterogeneity can be shown as diverse cell types (either basal-like or luminal) within more tumors. The predominance of the type of tumor depends on the tumor cell type, either basal-like or luminal respectively. But the heterogeneity contributes largely to the variability of the tumor so that not all basal-like and luminal tumors look similar [34]. According to a retrospective study of 162 patients by Marina et al., intratumoral heterogeneity was reliant on the molecular subtype of BC and related to the frequency of lymph node metastasis and the number of positive nodes. There was an association between alveolar structures and lymph node metastasis in postmenopausal women. Figure 3 demonstrates intratumor and intertumor heterogeneity.

Intertumoral Heterogeneity

It can be described as a subgroup of cells in more than one tumor, demonstrating the basal-like cancer cells and luminal-type cancer cells also described in Figure 3. Kornelia et al. stated that intertumor heterogeneity can display different phenotypes from basal-like and luminal aspects within tumors and high variability can be seen from each type of tumor in basal-like or luminal neoplasms [33].

TNBCs and related markers in basal-like breast cancer tumors

Basal-like breast cancer is likely to be present in TNBCs and can also show expression of CK5/6 and EGFR, which are markers seen in hereditary tumors such as BRCA1 [40]. Moreover, it has shown high expression of molecular pathways such as phosphatidylinositol 3-kinase activity in basal-like tumors, which were linked with a worse prognosis compared to luminal-like tumors xenografts in animal models [41]. More factors are associated with poor prognosis besides CD44/CD24 and basal-like tumor features. According to Wang et al., the presence of greater than or equal to four lymph nodes has also been related to a higher rate of tumor recurrence and mortality in women with TNBC [42]. CD44+/CD24- has been associated with high levels of ALDH expression [43,44,20]. These markers have shown the capability for self-renewal in mammalian spheroid studies [45,46,47]. It has not been proven that CSCs originate from mutations in resident stem cells or quiescent resident cells [48]. Additionally, CD44+/CD24- show epithelial-mesenchymal transition (EMT) phenotype that has a significant capability for invasion and metastasis [49,50,20].

ALDHA1 in cancer stem cells and cancer tissues

In light of the recent cancer stem cell hypothesis, neoplasms have a small percentage of neoplastic cells and a major percentage of non-neoplastic cells [51,7]. The small amount of cancer stem cells or tumor-initiating cells has been determined through xenograft transplantation assays to have tumorigenic activity [52,20]. Many isoforms of ALDH such as ALDH1A1, ALDH1A2, and ALDH1A3 have been discovered, which have an important role in retinoic acid formation through oxidation of all-trans-retinal and 9-cis-retinal. This retinoid acid has been shown to be associated with the stemness of cancer stem cells as well as normal tissue stem cells [53,54].

An inverse relationship between reactive oxygen species (ROS) and ALDH activity has been established [55]. A higher ALDH level results in lower levels of ROS levels, which was seen in some malignancies, suggesting increased antioxidant activity [56]. Evidence has indicated that ALDH1 is involved, and particularly its isotype ALDHA1 functioning as a cancer stem marker that can be used to enrich tumor-initiation subpopulations from various cell lines and primary tumors [57,58,59]. Unexpectedly, it has been described that those high levels of ALDHA1 expression did not relate to highly malignant phenotypes and poor clinical outcomes in some types of cancers [60]; maybe it depended on the method used to scope ALDH1A1 expression (i.e., flow cytometry or immunohistochemistry with an ALDHA1 antibody), sample size, or types of tissues [61,62].

The difference between normal tissue stem cells and cancer stem cells

Normal tissue stem cells and cancer stem cells contribute to many mechanisms associated with stemness, but there are differences between normal stem cells and cancer stem cells. Currently, the differences are disputable with regard to normal tissue stem cells and cancer stem cells [63]. Firstly, normal stem cells are usually oligo- or multipotent; it is unsure if cancer stem cells can become multiple differentiated cell types [60]. Secondly, it is disputable whether normal cellular precursors of cancer stem cells are real normal tissue stem cells. More variables are involved in cancer stem cell models that have been studied, such as the local microenvironment, i.e., niche and normal stem cells. In normal stem cells, the neighboring microenvironment is normal; however, CSCs usually have abnormal microenvironments, such as hypoxia, profound inflammation, and low nutrient conditions [60].

High levels and expression of ALDHA1 in breast cancer

In studies in vitro, it has been shown that ALDHA1 positivity in breast cancer cells could initiate tumor invasion and tumor metastasis in mice xenografts [64]. Also, ALDH1A1 has been considered a predictive factor in early metastasis and decreased survival in inflammatory breast cancer [60]. Ginestier et al. [58] demonstrated that increased expression of ALDH1A1 mRNA was associated with poorer OS in women with breast cancer. Consequently, ALDH1A1 was the only ALDH1 isoenzyme that could be used as a maker to predict unfavorable survival in breast cancer patients [65]. Therefore, ALDHA1 could be a crucial factor to generate ALDH1 activity in breast cancer. Its high expression of ALDHA1 mRNA was related to poor OS in breast cancer patients [60]. Then, a positive ALDH1 display can be a predictive marker. A meta-analysis showed that ALDH1A1 could indicate a poor prognosis in women with breast cancer [66]. In conclusion, ALDH1A1 is a promising cancer stem cell marker and a significant predictor of progression and poor survival.

Limitations

In this study, the authors mainly focused on retrospective studies where the sample size was small. Most of the techniques used to identify markers were from tissue microarray that can be sensitive to human errors such as localization or layout of the core for studying, storage, and multiple punches of the tissue to ensure adequate representations of the samples examined, especially for the heterogeneous tissues in the tumor. This study mainly used two markers: CD44+/CD24- cell stem markers and ALDH/ALDH1. Many other studies have incorporated other attributes such as markers and cytokines related to stem cells and tumor microenvironment variables, including micronutrients, inflammation markers, and hypoxia.

## Conclusions

This review reports that the CD44/CD24 and high levels of ALDHA1/ALDH in the BCSC phenotype have been involved as a prognostic element for TNBC. Women who present at the onset of the disease with a negative hormonal profile (ER/PR and HER2) marker in addition to CD44+/CD24- phenotype expression have a poor prognosis and unfavorable clinical outcomes. A majority of TNBC cancers are associated with a basal-like type. Still, it is vital to keep in mind the classifications given by Lehmann et al. who divided TNBC into six molecular subgroups: immunomodulatory (IM), mesenchymal (NI), mesenchymal stem-like (MSL), luminal androgen receptor (LAIR), and two basal-like subtypes (BL1 and BL2), and the classification according to Burstein et al. who used DNA profiling to identify TNBC subgroups such as cluster 1: luminal AR (LAR), cluster 2: mesenchymal (MES), cluster 3: basal-like immunosuppressed (BLIS), and cluster 4: basal-like immune-activated (BLIA). These molecular divisions can help to categorize breast tumors in TNBC, which has been associated with the presence of BCSC markers and high heterogeneity in breast cancer tumors showing a significant role in tumor features such as differentiation, vascular invasion, tumorigenesis, and metastatic potential. Moreover, stem cell markers such as CD44/CD24 and high levels of ALDHA1 can predict stemness in triple-negative tumor features; also, more than four lymph nodes are associated with unfavorable outcomes. Further detailed studies and clinical trials should be conducted to identify more markers related to molecular pathways associated with cancer stem cells, which can lead to ideal treatments for TNBC and hopefully better clinical outcomes.

## References

[1] Breast cancer. World Health Organization.26 Mar. . https://www.who.int/news-room/fact-sheets/detail/breast-cancer (2022). World Health Organization: breast cancer. https://www.who.int/news-room/fact-sheets/detail/breast-cancer.

[2] Schmadeka R, Harmon BE, Singh M (2014). Triple-negative breast carcinoma: current and emerging concepts. Am J Clin Pathol.

[3] Elsamany S, Abdullah S (2014). Triple-negative breast cancer: future prospects in diagnosis and management. Med Oncol.

[4] Burstein HJ (2013). Patients with triple negative breast cancer: is there an optimal adjuvant treatment?. Breast.

[5] Foulkes WD, Smith IE, Reis-Filho JS (2010). Triple-negative breast cancer. N Engl J Med.

[6] Prat A, Adamo B, Cheang MC, Anders CK, Carey LA, Perou CM (2013). Molecular characterization of basal-like and non-basal-like triple-negative breast cancer. Oncologist.

[7] Jordan CT, Guzman ML, Noble M (2006). Cancer stem cells. N Engl J Med.

[8] Ailles LE, Weissman IL (2007). Cancer stem cells in solid tumors. Curr Opin Biotechnol.

[9] Tirino V, Desiderio V, Paino F (2013). Cancer stem cells in solid tumors: an overview and new approaches for their isolation and characterization. Faseb J.

[10] Al-Hajj M, Clarke MF (2004). Self-renewal and solid tumor stem cells. Oncogene.

[11] Dean M, Fojo T, Bates S (2005). Tumour stem cells and drug resistance. Nat Rev Cancer.

[12] Bao B, Ahmad A, Azmi AS, Ali S, Sarkar FH (2013). Overview of cancer stem cells (CSCs) and mechanisms of their regulation: implications for cancer therapy. Curr Protoc Pharmacol.

[13] Camerlingo R, Ferraro GA, De Francesco F (2014). The role of CD44+/CD24-/low biomarker for screening, diagnosis and monitoring of breast cancer. Oncol Rep.

[14] Chuthapisith S, Eremin J, El-Sheemey M, Eremin O (2010). Breast cancer chemoresistance: emerging importance of cancer stem cells. Surg Oncol.

[15] Abraham BK, Fritz P, McClellan M, Hauptvogel P, Athelogou M, Brauch H (2005). Prevalence of CD44+/CD24-/low cells in breast cancer may not be associated with clinical outcome but may favor distant metastasis. Clin Cancer Res.

[16] Idowu MO, Kmieciak M, Dumur C, Burton RS, Grimes MM, Powers CN, Manjili MH (2012). CD44(+)/CD24(-/low) cancer stem/progenitor cells are more abundant in triple-negative invasive breast carcinoma phenotype and are associated with poor outcome. Hum Pathol.

[17] Lehmann BD, Bauer JA, Chen X, Sanders ME, Chakravarthy AB, Shyr Y, Pietenpol JA (2011). Identification of human triple-negative breast cancer subtypes and preclinical models for selection of targeted therapies. J Clin Invest.

[18] Burstein MD, Tsimelzon A, Poage GM (2015). Comprehensive genomic analysis identifies novel subtypes and targets of triple-negative breast cancer. Clin Cancer Res.

[19] Sherman L, Sleeman J, Herrlich P, Ponta H (1994). Hyaluronate receptors: key players in growth, differentiation, migration and tumor progression. Curr Opin Cell Biol.

[20] Al-Hajj M, Wicha MS, Benito-Hernandez A, Morrison SJ, Clarke MF (2003). Prospective identification of tumorigenic breast cancer cells. Proc Natl Acad Sci U S A.

[21] Ponti D, Costa A, Zaffaroni N (2005). Isolation and in vitro propagation of tumorigenic breast cancer cells with stem/progenitor cell properties. Cancer Res.

[22] Aigner S, Sthoeger ZM, Fogel M (1997). CD24, a mucin-type glycoprotein, is a ligand for P-selectin on human tumor cells. Blood.

[23] Baumann P, Cremers N, Kroese F (2005). CD24 expression causes the acquisition of multiple cellular properties associated with tumor growth and metastasis. Cancer Res.

[24] Khan S, Suryavanshi M, Kaur J (2021). Stem cell therapy: a paradigm shift in breast cancer treatment. World J Stem Cells.

[25] Akbarzadeh M, Maroufi NF, Tazehkand AP (2019). Current approaches in identification and isolation of cancer stem cells. J Cell Physiol.

[26] Lapidot T, Sirard C, Vormoor J (1994). A cell initiating human acute myeloid leukaemia after transplantation into SCID mice. Nature.

[27] Wicha MS (2007). Breast cancer stem cells: the other side of the story. Stem Cell Rev.

[28] Dick JE (2009). Looking ahead in cancer stem cell research. Nat Biotechnol.

[29] Chekhun SV, Zadvorny TV, Tymovska YO, Anikusko MF, Novak OE, Polishchuk LZ (2015). СD44+/CD24- markers of cancer stem cells in patients with breast cancer of different molecular subtypes. Exp Oncol.

[30] Li W, Liu F, Lei T (2010). The clinicopathological significance of CD44+/CD24-/low and CD24+ tumor cells in invasive micropapillary carcinoma of the breast. Pathol Res Pract.

[31] Giatromanolaki A, Sivridis E, Fiska A, Koukourakis MI (2011). The CD44+/CD24- phenotype relates to 'triple-negative' state and unfavorable prognosis in breast cancer patients. Med Oncol.

[32] Kim HJ, Kim MJ, Ahn SH (2011). Different prognostic significance of CD24 and CD44 expression in breast cancer according to hormone receptor status. Breast.

[33] Zavyalova MV, Denisov EV, Tashireva LA (2013). Phenotypic drift as a cause for intratumoral morphological heterogeneity of invasive ductal breast carcinoma not otherwise specified. Biores Open Access.

[34] Polyak K (2011). Heterogeneity in breast cancer. J Clin Invest.

[35] Dick JE (2003). Breast cancer stem cells revealed. Proc Natl Acad Sci U S A.

[36] Liu AY, True LD, LaTray L (1997). Cell-cell interaction in prostate gene regulation and cytodifferentiation. Proc Natl Acad Sci U S A.

[37] Honeth G, Bendahl PO, Ringnér M (2008). The CD44+/CD24- phenotype is enriched in basal-like breast tumors. Breast Cancer Res.

[38] Meyer MJ, Fleming JM, Ali MA, Pesesky MW, Ginsburg E, Vonderhaar BK (2009). Dynamic regulation of CD24 and the invasive, CD44posCD24neg phenotype in breast cancer cell lines. Breast Cancer Res.

[39] Marusyk A, Polyak K (2010). Tumor heterogeneity: causes and consequences. Biochim Biophys Acta.

[40] Nielsen TO, Hsu FD, Jensen K (2004). Immunohistochemical and clinical characterization of the basal-like subtype of invasive breast carcinoma. Clin Cancer Res.

[41] Moestue SA, Dam CG, Gorad SS (2013). Metabolic biomarkers for response to PI3K inhibition in basal-like breast cancer. Breast Cancer Res.

[42] Wang H, Wang L, Song Y (2017). CD44+/CD24- phenotype predicts a poor prognosis in triple-negative breast cancer. Oncol Lett.

[43] Ginestier C, Hur MH, Charafe-Jauffret E (2007). ALDH1 is a marker of normal and malignant human mammary stem cells and a predictor of poor clinical outcome. Cell Stem Cell.

[44] Li W, Ma H, Zhang J, Zhu L, Wang C, Yang Y (2017). Unraveling the roles of CD44/CD24 and ALDH1 as cancer stem cell markers in tumorigenesis and metastasis. Sci Rep.

[45] Van Phuc P, Nhan PL, Nhung TH (2011). Downregulation of CD44 reduces doxorubicin resistance of CD44CD24 breast cancer cells. Onco Targets Ther.

[46] Bartucci M, Dattilo R, Moriconi C (2015). TAZ is required for metastatic activity and chemoresistance of breast cancer stem cells. Oncogene.

[47] Palomeras S, Ruiz-Martínez S, Puig T (2018). Targeting breast cancer stem cells to overcome treatment resistance. Molecules.

[48] Talukdar S, Bhoopathi P, Emdad L, Das S, Sarkar D, Fisher PB (2019). Dormancy and cancer stem cells: an enigma for cancer therapeutic targeting. Adv Cancer Res.

[49] Liu S, Cong Y, Wang D (2014). Breast cancer stem cells transition between epithelial and mesenchymal states reflective of their normal counterparts. Stem Cell Reports.

[50] Liu H, Patel MR, Prescher JA (2010). Cancer stem cells from human breast tumors are involved in spontaneous metastases in orthotopic mouse models. Proc Natl Acad Sci U S A.

[51] Reya T, Morrison SJ, Clarke MF, Weissman IL (2001). Stem cells, cancer, and cancer stem cells. Nature.

[52] Bonnet D, Dick JE (1997). Human acute myeloid leukemia is organized as a hierarchy that originates from a primitive hematopoietic cell. Nat Med.

[53] Marcato P, Dean CA, Giacomantonio CA, Lee PW (2011). Aldehyde dehydrogenase: its role as a cancer stem cell marker comes down to the specific isoform. Cell Cycle.

[54] Marcato P, Dean CA, Pan D (2011). Aldehyde dehydrogenase activity of breast cancer stem cells is primarily due to isoform ALDH1A3 and its expression is predictive of metastasis. Stem Cells.

[55] Ikeda J, Mamat S, Tian T (2012). Reactive oxygen species and aldehyde dehydrogenase activity in Hodgkin lymphoma cells. Lab Invest.

[56] Mizuno T, Suzuki N, Makino H (2015). Cancer stem-like cells of ovarian clear cell carcinoma are enriched in the ALDH-high population associated with an accelerated scavenging system in reactive oxygen species. Gynecol Oncol.

[57] Huang EH, Hynes MJ, Zhang T (2009). Aldehyde dehydrogenase 1 is a marker for normal and malignant human colonic stem cells (SC) and tracks SC overpopulation during colon tumorigenesis. Cancer Res.

[58] Ginestier C, Korkaya H, Dontu G, Birnbaum D, Wicha MS, Charafe-Jauffret E (2007). The cancer stem cell: the breast cancer driver (Article in French). Med Sci (Paris).

[59] Deng S, Yang X, Lassus H (2010). Distinct expression levels and patterns of stem cell marker, aldehyde dehydrogenase isoform 1 (ALDH1), in human epithelial cancers. PLoS One.

[60] Tomita H, Tanaka K, Tanaka T, Hara A (2016). Aldehyde dehydrogenase 1A1 in stem cells and cancer. Oncotarget.

[61] Tanei T, Morimoto K, Shimazu K (2009). Association of breast cancer stem cells identified by aldehyde dehydrogenase 1 expression with resistance to sequential Paclitaxel and epirubicin-based chemotherapy for breast cancers. Clin Cancer Res.

[62] Jiang F, Qiu Q, Khanna A (2009). Aldehyde dehydrogenase 1 is a tumor stem cell-associated marker in lung cancer. Mol Cancer Res.

[63] Gupta PB, Chaffer CL, Weinberg RA (2009). Cancer stem cells: mirage or reality?. Nat Med.

[64] Charafe-Jauffret E, Ginestier C, Iovino F (2010). Aldehyde dehydrogenase 1-positive cancer stem cells mediate metastasis and poor clinical outcome in inflammatory breast cancer. Clin Cancer Res.

[65] Morimoto K, Kim SJ, Tanei T (2009). Stem cell marker aldehyde dehydrogenase 1-positive breast cancers are characterized by negative estrogen receptor, positive human epidermal growth factor receptor type 2, and high Ki67 expression. Cancer Sci.

[66] Liu Y, Lv DL, Duan JJ (2014). ALDH1A1 expression correlates with clinicopathologic features and poor prognosis of breast cancer patients: a systematic review and meta-analysis. BMC Cancer.

